# Metabolic Signatures of the Exposome—Quantifying the Impact of Exposure to Environmental Chemicals on Human Health

**DOI:** 10.3390/metabo10110454

**Published:** 2020-11-10

**Authors:** Matej Orešič, Aidan McGlinchey, Craig E. Wheelock, Tuulia Hyötyläinen

**Affiliations:** 1 School of Medical Sciences, Örebro University, SE-701 82 Örebro, Sweden; matej.oresic@oru.se (M.O.); Aidan.McGlinchey@oru.se (A.M.); 2 Turku Bioscience Centre, University of Turku and Åbo Akademi University, FI-20520 Turku, Finland; 3 Division of Physiological Chemistry II, Department of Medical Biochemistry and Biophysics, Karolinska Institute, SE-171 77 Stockholm, Sweden; Craig.Wheelock@ki.se; 4 MTM Research Centre, School of Science and Technology, Örebro University, SE-701 82 Örebro, Sweden

**Keywords:** chemical exposure, disease biomarkers, exposome, human health, lipidomics, metabolomics, per- and polyfluoroalkyl substances

## Abstract

Human health and well-being are intricately linked to environmental quality. Environmental exposures can have lifelong consequences. In particular, exposures during the vulnerable fetal or early development period can affect structure, physiology and metabolism, causing potential adverse, often permanent, health effects at any point in life. External exposures, such as the “chemical exposome” (exposures to environmental chemicals), affect the host’s metabolism and immune system, which, in turn, mediate the risk of various diseases. Linking such exposures to adverse outcomes, via intermediate phenotypes such as the metabolome, is one of the central themes of exposome research. Much progress has been made in this line of research, including addressing some key challenges such as analytical coverage of the exposome and metabolome, as well as the integration of heterogeneous, multi-omics data. There is strong evidence that chemical exposures have a marked impact on the metabolome, associating with specific disease risks. Herein, we review recent progress in the field of exposome research as related to human health as well as selected metabolic and autoimmune diseases, with specific emphasis on the impacts of chemical exposures on the host metabolome.

## 1. Introduction

It is currently widely recognized that combinations of environmental factors, interacting further with genetic factors, play crucial roles in human health and disease. Indeed, a majority of genome-wide-association studies (GWAS) have detected only relatively minor effects of common genetic variants on the incidence of most non-communicable diseases (NCDs) [1,2,3].

The exposome concept was first coined by Christopher P. Wild in 2005, when describing “*the totality of human environmental exposures from conception onwards, complementing the genome*” [3]. The exposome concept includes lifetime exposure, combining exogenous chemicals with genetic and other external factors that generate further molecular products inside the body and thereby may present threats to human health [1,4,5,6]. In this context, environmental factors comprise the full suite of general external, specific external and internal exposures [7,8]. General external exposures include the broader socioeconomic environment such as social capital, education level, built environment, urban-rural environment, and climate factors. Specific external exposures include an individual’s external factors such as stress, specific contaminants, diet, physical activity, substance habits, allergens and infections. Internal exposures, on the other hand, include biological factors such as metabolism, the immune system, and the gut microbiome. It is understandably challenging to quantify these hyper-variable interdependent environmental and lifestyle factors because exposures are individual, have multiple sources with high complexity, and are dynamic.

Herein, we review recent progress in the field of exposome research as related to human health and selected metabolic and autoimmune diseases, with specific emphasis on the impacts of the “chemical exposome” (exposures to environmental chemicals) on the host metabolome.

## 2. Exposomics Approach to Study Health and Disease

Current chemical exposure assessments are still limited, with most of the reported studies covering a small fraction of chemicals to which humans are exposed. Any hypothetical total number of chemicals in the world is unknown, but over 140,000 and 86,000 chemicals have been registered for use in Europe and the USA, respectively [9]. This demonstrates the enormous number of chemicals, pollutants, and contaminants that humans may potentially be exposed to, the majority of which lack substantive data to perform comprehensive risk assessment. In addition, the assessment of exposure health risks is primarily based on studies involving individual chemicals or within chemical classes, or the use of various toxicological indices, which are themselves typically developed from studies using only single chemicals. This does not give a realistic overview of health risks, because mixtures of various chemicals may have a highly different impact on health than when taken in isolation. For instance, there is evidence suggesting that specific endocrine-disrupting chemicals compete with each other and with endogenous estrogens for access to metabolic enzymes and that this may lead to increased bioavailability of specific harmful chemicals [10]. Exposure to numerous chemicals could thus potentially have adverse effects at doses much closer to typical human exposures than previously assumed. Studies of chemical mixture exposure in humans (and children in particular) are therefore strongly warranted, when conducting quantitative risk assessments and determining regulatory exposure limits. While the main routes of human exposure to environmental chemicals for the general population include food, house dust, and drinking water in infants, occurrence of these compounds is attributed to placental transfer during fetal development, and breastfeeding. Moreover, in childhood, exposure via house dust can be an additional major source. In addition, exposure via airways and skin can be relevant to specific chemicals [11,12].

It should be emphasized that while screening pollutant profiles and their occurrence levels in humans or biota is important, this is not the most efficient way to characterize the health impacts of chemical exposures. Furthermore, the presence of a pollutant does not necessarily imply health impact. Not only does the screening approach require an enormous effort, but it does not account for individual variation in both the exposure and various critical biological factors. Exposure profiles are known to be strongly related to race/ethnicity, age, body mass index (BMI), as well as with several other social, environmental, and individual factors [13]. Furthermore, pollutant profiles in humans, if considered in isolation, would provide, at best, only incomplete health risk information compared to an analysis of chemical mixtures. A more efficient approach, therefore, is to employ a comprehensive analysis that covers both exogenous and endogenous compounds, including their metabolites. This approach, combined with health outcome data and accompanying metabolomics, i.e., comprehensive characterization of small molecule metabolic products (metabolites), can provide a tool for the identification of early biomarkers linked to exposure and, in turn, offer new opportunities in exposome research [14]. Measuring comprehensive chemical exposure profiles and investigating their respective associations with determinants of health status will enable an in-depth analysis of the links between these and result in more reliable conclusions.

The number of studies related to the exposome has markedly increased over the past decade [15,16,17,18,19,20,21,22,23,24,25]. A large number of epidemiological studies have sought to identify the associations between exposure to environmental chemicals and different chronic diseases, such as diabetes, cancer, and obesity [23,26,27,28,29,30,31,32]. Exposure studies using animal models have identified potential mechanisms, elucidating the impacts of various exposures on specific biochemical pathways. Chemical exposome studies are also emerging, integrating the profiling of exogenous and endogenous compounds with their impact on adverse health outcomes [33,34]. It should be noted, however, that there is a risk for obtaining false positive exposome-health associations due to the complex correlation structure of the exposome. It is very challenging (1) to efficiently untangle the exposures that causally impact specific health outcomes from spuriously associated exposures and (2) to identify synergistic effects between exposures. Therefore, it is crucial to give a careful consideration to both in the selection of appropriate statistical methods as well as to the interpretation of the results [35].

## 3. Analytical Methodologies

Characterization of the exposome requires the application of a diverse range of analytical techniques [15,16,17,36,37]. The methodologies required for exposome analysis as related to the study of chemical exposures include:

1. Analytical techniques for comprehensive chemical profiling (exogeneous and endogenous compounds);
2. Effect-directed analysis of the drivers of toxicity;
3. Advanced bioinformatics methods, in order to integrate the highly complex data and to identify effect-based markers of exposure.

Early exposome studies relied on the so-called “bottom-up approach”, where the main sources of exposure (e.g., water, food, air) were screened for pollutants, followed by further analyses. The opposite, alternative approach is to start the screening in human samples, i.e., using the so-called “top-down approach”, where chemical profiles are first associated with health status, and only later on the possible sources of exposures are linked with adverse health outcomes. The use of a “*meet in the middle*” (MITM) approach, which combines bottom-up and top-down approaches, has also been increasing [38]. This approach includes measuring intermediate biomarkers such as metabolites or other “omics” biomarkers, and retrospectively relating them to measurements of external exposure as well as prospectively to a specific health outcome. Specifically, the aim is to comprehensively characterize phenotypes in a human cohort setting, using multi-omics techniques (e.g., metabolomics, proteomics and transcriptomics) in order to identify specific adverse pathways affected by exposures and consequently driving disease risk. In the next step, in silico exploitation of (toxicological) databases and chemical bioactivity from high-throughput screening assays, reporter gene assays and docking studies can be used for the identification of pathway-associated exogenous and endogenous chemicals.

### 3.1. Analytical Methods

**Figure 1:**
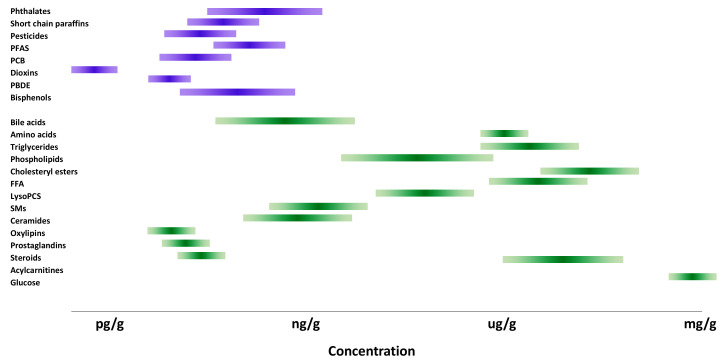
Plasma concentration ranges of the most common persistent organic pollutants (POPs) and metabolites.

In order to comprehensively characterize the chemical profiles of the samples, analytical methods should cover both endogenous compounds (metabolites) as well as exogenous compounds, such as environmental pollutants. However, because the levels of many key metabolites are substantially higher than those of the environmental chemicals of concern (Figure 1), it is usually not possible to analyze all compounds within the same analytical protocols. In both cases, liquid or gas chromatography (LC, GC), combined with mass spectrometry (MS), is commonly used. Both LC and GC, combined with high-resolution mass spectrometry (HRMS) analyzers, including Orbitrap, Fourier transform ion cyclotron resonance (FT-ICR), time-of-flight mass spectrometry (TOF MS), and hybrid MS configurations, such as quadrupole-TOF (Q-TOF), ion-trap-TOF (IT-TOF), or quadrupole-Orbitrap (Q-Orbitrap) are used in exposome studies. In LC-based methods, soft ionization techniques such as electrospray or atmospheric chemical ionization are typically applied, while in GC-based methods, both hard (electron impact) as well as soft ionization (chemical ionization, atmospheric chemical ionization) are used. These methods can be applied with different acquisition modes including data-dependent acquisition (DDA), data independent acquisition (DIA) and all ion fragmentation (MS^all^). A combination of targeted, non-targeted, as well as suspect screening approaches, is typically applied in exposome analysis. Often, the environmental chemicals are analyzed with either target or suspect screening modes, while in metabolomics, non-targeted acquisition predominates. Specific metabolites may, however, require targeted strategies, either due to their low levels or their instability.

There are several, established workflows described for untargeted metabolomics, most of which use at least two methods to cover both polar and non-polar metabolites. A majority of the methods are based on high-resolution mass spectrometric methods, combined with either LC or GC after very simple sample preparation, typically either by simple protein precipitation or by liquid extraction [17,39,40,41,42,43]. For the characterization of environmental pollutants, either in targeted, untargeted or suspect screening mode, more involved sample preparation methods are often required to remove the interfering compounds (e.g., abundant lipids). This is necessary in order to obtain sufficient sensitivity for the analysis. Larger amounts of sample are typically needed for the analysis of environmental contaminants as compared to metabolites and sample clean-up is required (e.g., liquid extraction, solid-phase extraction, phospholipid depletion). Most of the traditional methods are targeted analyses, again, mainly to obtain sufficient sensitivity, and the most common methods are based on GC–MS and LC–MS/MS. Recently, untargeted analysis and suspect screening approaches have been developed for the screening of the exogenous pollutants [44,45,46,47,48]. 

Reliable identification in the suspect screening and in untargeted analysis remains highly challenging. Large spectral libraries are available for GC-based methods and, in recent years, a major effort has been undertaken to compile LC-based spectral libraries. Unsupervised and supervised machine learning approaches can be utilized in the identification of unknown compounds. For example, using an unsupervised algorithm, MS2Analyzer combines structural information inherent to product ions and their fragments, neutral losses and isotopic ratios, with literature-derived neutral loss/substructure pairs to detect the presence of the same or similar substructures [49]. Supervised machine learning classification methods can be applied for the detection of specific substructures or structural neighbors according to the presence of predefined substructures and classification of unknowns accordingly [49,50,51] or for the determination of spectral features. Direct structure elucidation methodologies can also be applied using general fragmentation rules using, e.g., the publicly available MS-FINDER [52] software. However, fragmentation can often be unpredictable, and structurally similar compounds do not always generate similar fragments. These approaches do generally work, however, for specific types of compounds, such as lipids. Several approaches using various indirect structure elucidation approaches as well as in silico spectral prediction approaches have been developed [53,54,55,56,57]. In untargeted analyses, it is possible to utilize the unique mass spectral features of, e.g., halogenated compounds, so that it is possible to screen these compounds based on the mass spectrum. Particularly in GC–MS based methods, it is possible to use compound classification based on mass-spectral fragmentation patterns. These procedures have been developed for various environmental samples and for metabolomics [58,59,60], although they have rarely been applied in exposome analysis to date. 

### 3.2. Data Analysis

Increasing levels of detail and increased understanding of the interconnected nature of various levels of biological organization form both the basis and a key objective of taking the holistic approach that is systems medicine. This is of particular relevance in exposome research, because complex and often subtle effects with subsequent knock-on effects occur up and down the different levels of organization within organisms, including humans. Appropriate methods are therefore required to meaningfully tease apart changes of interest across multiple levels of high-throughput data, bringing together exposure data (being often high-dimensional) with various other strata of data (e.g., metabolomic, proteomic, lipidomic, transcriptomic or otherwise).

A first and crucial step in data analysis for exposome and metabolomics research is pre-processing of the data (e.g., via log transformation, scaling to zero mean and unit variance in the case of metabolomics data) rendering the measured levels of metabolites/chemical exposure of interest amenable to correlation, or other associative, analyses to search for relationships.

In exposome analysis, therefore, both appropriate pre-processing and data integration techniques are crucial, both in the case of (1) integrating exposome data with high-dimensional, multi-omics data, and (2) when investigating the effects exposure to complex chemical mixtures, in order to identify biomarkers of said exposure. Further, given that exposure data from the general populace is inherently heterogenous, this presents a further layer of complexity that also must be dealt with, either at the level of study design, or with appropriate statistical or machine learning methods.

Regarding exposure data in particular, it is now well-recognized in the field that, when translating the impacts of chemical exposures to measurable health outcomes, one needs to model such exposures as mixtures, rather than only as single effects and doses. This poses methodological challenges that are still being debated in the field [61]. Typically, the impact of exposure to mixtures of agents on specific outcome variables is modelled with a regression approach. Many statistical/machine learning methods lend themselves to this purpose, and these are extensively reviewed and assessed by Agier et al. [61]. Among these, particularly Bayesian kernel machine regression (BKMR) is a popular choice for estimating the health effects of chemical mixtures [62], but, as is also the case for most other methods, its use is limited to estimation of an effect on a single outcome variable at a time, rendering it unsuitable for application to complex phenotypic data (e.g., multi-omics data or where more than one clinical outcome may be affected by exposure to the mixture). 

In the case of complex, multi-variate or multi-modal outcomes, one data analysis strategy is to combine regression approaches with network analysis [34]. In such an approach, chemical exposure data, along with multi-modal phenotypic data (dimensionality reduced, e.g., by model-based clustering [63], if necessary or applicable) are brought together and analyzed by first applying partial correlation network analysis. This involves first the crucial step of rejecting likely spurious correlations, e.g., through the calculation of non-rejection rates as available in openly available packages for the R statistical programming language, such as the qp-graph package [64]. Based on this analysis, a network of interacting components between (1) the exposome, (2) intermediary layer(s) of (potentially also dimensionality-reduced) data (e.g., metabolomics or other -omics), and (3) the various clinical outcomes of interest can be projected and subsequently both analyzed and meaningfully interpreted holistically. Further, “connected” outcome variables of interest can then be selected, and regression analyses performed linking chemical mixtures with outcome variable(s) of interest (e.g., by iterative ridge regression modelling using bootstrapping [34]). Such an examination of the network can (1) help identify key phenotypic variables of interest by removing spurious associations, and (2) provide predictive and relative contributions of individual chemicals towards the variable(s) of interest.

Further, with the striking rise in the application and development of machine learning methods, great potential exists for its application to exposome research, enabling robust interrogation of large, multi-omics datasets to ascertain key factors affecting real-world health outcomes in data from both purely experimental settings as well as from the harvesting of population data.

With increasing computational power and software available to researchers, the possibility now exists for thorough mining of exposome data to link complex mixtures of exposures to not only clinical outcomes, but to assist in the elucidation of key mechanisms occurring at the various biological levels of organization and information flow between exposure and health effects. Appropriately, methods such as multi-layer (deep) artificial neural networks (ANNs) are proving to be of striking utility across a plethora of fields for analyzing high-dimensional, heterogeneous data for purposes of classification (e.g., health outcome) and regression type analyses (e.g., exposure effect on markers of interest). ANNs and associated deep learning methods currently suffer from issues of interpretability and are difficult to apply to the development of simpler clinical tests; other machine learning methods, such as the random forest, can currently provide clearer answers regarding the importance of the features that they use and divulge thresholds by which the models make their best-performing classification methods, forming the basis of direct translation to clinical testing. Machine learning and its application to metabolomics and multi-omics data are reviewed in detail elsewhere [65,66].

The ability to generate a meaningful, robust and multi-layered overview of biological effects from exposure to a mixture of agents addresses an unmet need in exposome research. This is a notable step for the field, clearly showing, for example, networks of interacting factors from heterogeneous, multi-omics data for downstream analysis, biomarker discovery and toxicity threshold testing of real-world mixtures of agents, which may behave markedly differently from more simplistic approaches that take such exposures as single factors.

## 4. Metabolic Markers of Exposure to Environmental Chemicals

**Figure 2:**
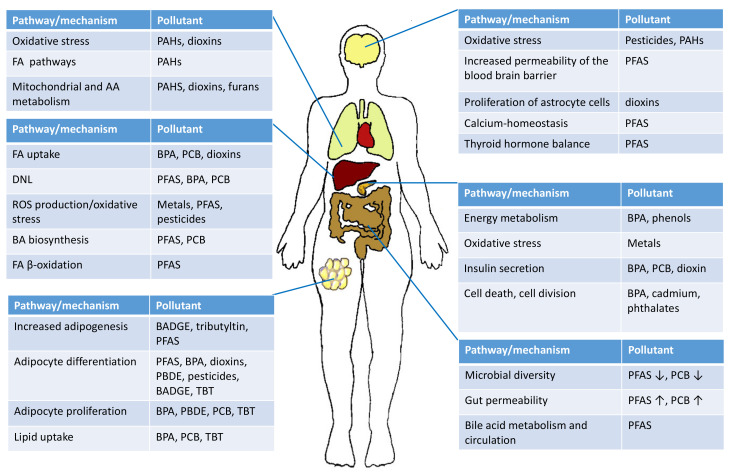
Reported impacts of exposure to environmental chemicals on metabolism.

The metabolome in exposome studies can be considered as an intermediate phenotype, linking exposures with health status [67]. Metabolic changes observed in exposome studies may thus provide clues about the changes in adverse outcome pathways, potentially linked to specific diseases (Figure 2). Whilst the number of studies wherein metabolomic profiles are associated with exposures in human cohorts is increasing, their overall number remains limited, providing information mainly only on specific chemical groups [24,25]. In addition, many of these studies involve only a small number of participants, using analytical methods with limited coverage of the metabolome. On the other hand, the challenge with in vivo and in vitro models is, however, that often the doses used in the experiments may not accurately reflect the true environmental concentration levels. Below, we review current knowledge about the impacts of chemical exposures on human metabolome.

### 4.1. Lipid Metabolism in Liver and Adipose Tissue

Several environmental chemicals have been found to impact lipid metabolism, including metals, polycyclic aromatic hydrocarbons (PAHs), per- and polyfluoroalkyl substances (PFASs) and polychlorinated biphenyls (PCBs). 

At the organ level, the two main organs involved in lipid metabolism are the liver and adipose tissue (AT). The liver is the largest and most metabolically complex organ in the human body, while AT is the key regulator of energy balance and nutritional homeostasis [68]. Adipocytes also regulate body weight, are an important site for the synthesis of estrogen and store steroid hormones in addition to playing a role in immune responses. AT can store a variety of hydrophobic chemicals, in particular persistent organic pollutants (POPs), and thus it constitutes a low-grade internal source of stored POPs, leading to continuous exposure in other tissues [69,70]. Moreover, xenobiotic compounds may alter AT functions, increase AT inflammation, and/or modulate differentiation of AT precursor cells [70]. 

Several pollutants have been shown to significantly alter the function (gene expression, hormone secretion) of white AT, AT mass (adipocyte number and/or volume), or body weight in animal models after developmental exposure [71,72,73,74,75]. In particular, tributyltin (TBT), phthalates, bisphenol A (BPA), diethylstilbestrol (DES), polyaromatic hydrocarbons (PAHs) and parabens have been shown to possess obesogenic properties [76,77,78,79]. These compounds can act through several pathways that promote adipogenesis and lipid accumulation. For most compounds classified as obesogens, prenatal exposure results in an increased number of adipocytes [80]. The main mechanism involved in adipogenesis acts through PPARγ function.

PCB153 has been shown to induce substantial alterations in levels of glycerophospholipids and sphingolipids in vitro [81,82]. Similar results were also observed in human studies [83]. Short-chain chlorinated paraffin studies have been shown to impact lipid metabolism in vitro through stimulation of β-oxidation of unsaturated fatty acids and long-chain fatty acids [84].

Metal exposure, such as to cadmium, lead or arsenic, has been suggested to cause increased lipid peroxidation [18,85,86,87]. Combined exposure to lead, cadmium and arsenic showed disturbances in energy metabolism, more precisely, changes in lipid fraction, unsaturated lipids and in the level of amino acids suggesting perturbation of lipid metabolism and amino acid metabolism [87]. Exposure to PAHs has been shown to have a marked impact on urinary metabolic profiles, and the metabolic outcomes of PAH exposure were generally associated with metabolites related to lipid metabolism, indicative of an oxidative stress response [88]. Chlorinated compounds such as dioxins, PCBs, organochlorine insecticides and trichloroethylene (TCE) have been shown to trigger similar metabolic changes as in PAHs and metals, but generating an even more substantial impact on lipid metabolism, including cholesterol metabolism, sphingolipid metabolism and bile acid (BA) biosynthesis, confirming their potential to induce chronic diseases such as atherosclerosis, diabetes or obesity [89,90,91,92].

A recent animal model study showed that dichlorodiphenyldichloroethylene (DDE) exposure leads to down-regulation of phosphatidylcholines (PCs), phosphatidylethanolamines (PEs), phosphatidylserines (PSs), and up-regulation of diacylglycerols (DGs), while triacylglycerols (TGs) were found both increased and decreased as a result of exposure, depending on the specific molecular species in question [93]. The study also indicated a potential role for gut microbiota–lipid interactions, as 17 bacterial species associate with lipids with notable correlations (*Bacteroidetes*, *Firmicutes*, *Proteobacteria* and *Tenericutes*, and DG, PC, PE and TG). 

In a Swedish cohort of elderly subjects, circulating levels of p,p′-DDE and hexachlorobenzene (HCB) levels were linked to a set of lipid-related metabolites involved in key metabolic processes such as cell signaling, energy regulation, and membrane composition (e.g., fatty acids and different classes of glycerophospholipids) [25]. The study also suggested that there may be differences in the lipid pathways impacted by the two organochlorine pesticides. Another study showed similar associations between the levels of DDE, β-HCH, HCB, PCBs and lipids, particularly in specific sphingolipids and glycerophospholipids [83]. The limitations of both of these studies was that the metabolomics analyses did not cover TGs and the most polar metabolites.

PFASs were also found to have an impact on lipid metabolism and energy metabolism [94,95]. In a recent study of 1000 elderly people in Sweden, 15 metabolites, predominantly from lipid pathways, were associated with levels of PFASs, following adjustment for sex, smoking, exercise habits, education, energy, and alcohol intake [24]. Perfluorononanoic acid (PFNA) and perfluoroundecanoic acid (PFUnDA) were strongly associated with multiple glycerophosphocholines and fatty acids, including docosapentaenoic acid (DPA) and docosahexaenoic acid (DHA) [24]. However, as dietary data were not available, some of the associations may be related to dietary factors, such as the intake of fish products, given that one of the main sources of PFAS in the Nordic population is fish intake, which has a somewhat similar impact on human levels of polyunsaturated fatty acid (PUFA)-containing lipids. These results indicate that the different PFASs evaluated were associated with distinctive metabolic profiles, suggesting potentially different biochemical pathways in humans. Perfluorooctanoic acid (PFOA), a widely used PFAS, was also found to be significantly associated with elevated uric acid in several studies [24,96]. Uric acid is an important metabolite in purine metabolism, and several epidemiologic studies, supported by studies in animal models, indicate that elevated uric acid is a risk factor for hypertension and possibly an independent risk factor for stroke, diabetes, and metabolic syndrome [97,98].

### 4.2. Bile Acids

BAs are metabolites that facilitate the digestion and absorption of lipids in the small intestine and they are also important metabolic regulators involved in the maintenance of lipid and glucose homeostasis [99,100]. BA metabolism is also closely linked with gut microbiota, which plays an essential role in the deconjugation of primary BAs and secondary BA synthesis. Exposure to POPs, such as PFAS and PCBs, has been shown to result in changes in the composition of the gut microbiota [101,102]. Several pollutants have shown to modulate BA metabolism, including PFAS, dioxins, and PCBs [95,102,103,104,105].

**Figure 3:**
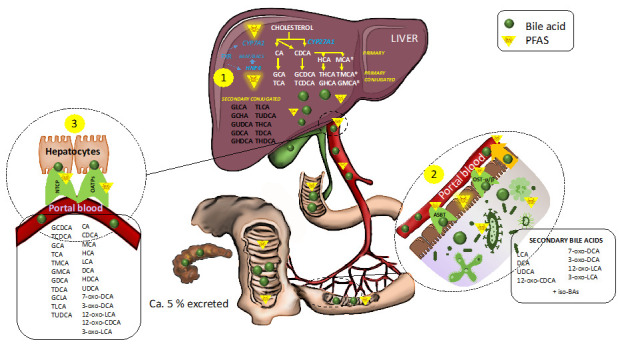
Bile acid biosynthesis and enterohepatic circulation, and the impact of polyfluoroalkyl substances (PFASs) on bile acid (BA) metabolism. (1) In the liver, the classic BA synthesis pathway is initiated by cholesterol 7α-hydroxylase (CYP7A1) which is downregulated by PFAS. The alternative BA synthesis pathway is initiated by CYP27A1 to synthesize primary bile acids, CA and CDCA, in hepatocytes. CDCA can be further converted to HCA and MCA in the liver. CYP7A1 is downregulated by PFAS. Bile acids are conjugated to the amino acids taurine or glycine before being released into the intestine. HNF4α, which plays a central role in bile acid conjugation by direct regulation of VLACSR and BAAT, can be suppressed by PFAS. (2) BAs are recovered into portal blood through a combination of passive absorption in the proximal small intestine, active transport via apical bile salt transporter (ASBT) in the distal ileum, and passive absorption in the colon and via organic solute transporter α/β (OSTα/β). Perfluorooctanesulfonic acid (PFOS) can also be transported by ASBT and OSTα/β. Furthermore, PFAS can increase the permeability of the gut, thus impacting the passive transport pathway of BAs. In the colon, BAs are also deconjugated by bacterial bile salt hydrolase and are 7α-dehydroxylated by bacterial 7α-dehydroxylase to form secondary BAs. PFAS can modify gut microbial composition and thus impact microbial BA formation. (3) BAs are eventually recycled from portal blood back to hepatocytes via Na-taurocholate co-transport peptide (NTCP) and the sodium-independent organic anion transporting polypeptide (OATP). PFASs also utilize the NTCP and OATP transporter. The majority (90–95%) of BAs secreted into the small intestine are actively reabsorbed in the terminal ileum and circulate back to the liver while ca. 5% are excreted via feces.

PFAS can have an impact on BA metabolism at several levels (Figure 3). For example, PFAS can inhibit 7-alpha-hydroxylase (CYP7A1), which catalyzes the first and rate-limiting step in the formation of BAs from cholesterol [30,106]. This may lead to increased re-uptake of BAs, which would generate negative feedback loops via the farnesyl-X-receptor (FXR) and subsequently reduce their de novo synthesis. PFOA also inhibits the function of hepatocyte nuclear factor 4α [107], which plays a central role in the regulation of BA metabolism in the liver and is linked to both the synthesis and conjugation of primary BAs. The liver clears most BAs via sodium taurocholate co-transporting polypeptide (NTCP), and several PFAS are also substrates for human NTCP [95]. PFAS exposure also alters the composition of the gut microbiota [101,102,103,108,109,110], which, in turn, can cause alteration in the pool of secondary BAs. Associations observed between PFAS and BAs are potentially important for our understanding of cardiometabolic diseases, given that BA metabolism is known to play a role in the pathogenesis of type 2 diabetes (T2D), atherosclerosis and non-alcoholic fatty liver disease (NAFLD) [111]. 

PCB exposure has been shown to modify the gut microbiota composition also and modulate BA homeostasis in conjunction with host BA processing genes in a dose- and bio-compartment-specific manner [103]. Current studies also indicate that PCBs modulate signaling within both neurons and epithelial cells, supporting the hypothesis that exposures may detrimentally impact the gut–brain axis [110].

### 4.3. Amino Acid Metabolism

Metals, plasticizers and other organic pollutants have been associated with altered amino acid metabolism. Exposure to cadmium, lead and arsenic has been suggested to cause various oxidative stress-related effects, including the depletion of antioxidants, accelerated muscle proteolysis, elevated activity of UDP-glucosyltransferases (UGTs) [18,85,86,87]. Specifically, cadmium exposure has been associated with metabolites related to amino acid metabolism, galactose metabolism, purine metabolism, the creatinine pathway as well as with steroid hormone biosynthesis [85]. Combined exposure to lead, cadmium and arsenic showed perturbation of the metabolism of amino acids [87]. Short-chain paraffins have been shown to disturb glycolysis and amino acid metabolism, leading to the up-regulation of glutamate metabolism and the urea cycle [84].

BPA exposure, as summarized in a recent review [112], is associated with alteration of branched chain amino acid metabolism, aromatic amino acids metabolism and sulfur-containing amino acid metabolism. Particularly, alterations to phenylalanine metabolism, tryptophan metabolism, tyrosine metabolism, lysine degradation, and arginine biosynthesis has been observed in female infants while male infants were less affected [113].

Exposure to PAHs has been associated with metabolites related to amino acid and purine metabolism, indicative of an oxidative stress response [88]. Altered amino acid metabolism has also been associated with PFAS exposure, particularly with regards to tyrosine metabolism [114]. Another study identified a positive association between PFAS exposure and phosphoethanolamine, tyrosine, phenylalanine, aspartate and creatine, and inverse association with betaine [115].

Altered amino acid metabolism is linked with several diseases, including diabetes, obesity and NAFLD. Increased levels of aromatic amino acids, such as tyrosine and phenylalanine, have been consistently found to be closely associated with hyperglycemia, insulin resistance and risk of type 2 diabetes [116,117,118,119].

### 4.4. Energy Metabolism and Oxidative Stress

Exposure to environmental chemicals, particularly to PAHs and metals, has shown to increase reactive oxygen species (ROS)—mediated oxidative stress in several studies [120,121]. Similarly, environmental chemical exposure has been shown to cause disruption of the tricarboxylic acid (TCA) cycle, thus impairing mitochondrial function and energy production [122].

Exposure to airborne pollution, including black carbon, carbon monoxide, nitrogen oxides and fine particulate matter, which most likely contains a large number of different organic contaminants, was linked with aggravation of inflammatory and oxidative stress-related pathways, including leukotriene and vitamin E metabolism [123].

Metabolic changes induced by phthalates affect both anti-oxidant mechanisms (mitochondrial beta-oxidation, amino acid metabolism) and disrupt prostaglandin-regulated pathways [124,125,126]. A recent study further showed that exposure to mono-n-butyl phthalate (MnBP) was associated with being overweight/obesity in children and elevated MnBP concentrations in urine correlated with global urine metabolic abnormalities as characterized by disrupted arginine and proline metabolism, increased oxidative stress and fatty acid re-esterification [126].

### 4.5. Impact of Environmental Exposure on Metabolome via Gut Microbiota

In addition to direct impacts of exposures on host metabolism, the exposures may also alter host metabolism indirectly, e.g., via gut or even skin microbiota. Exposure to environmental pollutants may cause adverse changes in the composition of the gut microbiota, resulting in gut dysbiosis. Moreover, such exposure can even alter the metabolic capacity of the gut microbiota, affecting the production of bacterial metabolites, which, in turn, may lead to adverse health effects in the host. However, many alterations of the gut microbiome composition can be secondary consequences of toxic effects acting on other organs and organ systems, and as such these effects should not be considered as only a direct effect on the gut microbiota.

A recent review by Jin et al. summarized the impact of different pollutants on the gut microbiota [127], showing that exposure can lead to various alterations in the gut microbiota favoring the growth of pathogenic bacteria whilst proving deleterious to beneficial bacteria. For example, a recent murine study showed that DDE induces gut dysbiosis as indicated by way of an increased *Firmicutes*-to-*Bacteroidetes* ratio, which may impact energy harvest efficiency [93]. A study in humans, on breast-fed infants, showed that POP exposure again impacted the gut microbiota, specifically, both PBDE-28 and the surfactant perfluorooctanesulfonic acid (PFOS), another widely used PFAS, were associated with reduced microbiome diversity [108]. Moreover, toxicants in breast milk affected microbiome functionality, explaining over 30% of variance in the levels of two short-chain fatty acids (acetic and propionic acids). Alterations in bacterial metabolism due to exposure have also been shown to cause bile acid dysmetabolism [128,129]. Chemical exposure may also negatively affect epithelial cells and so give rise to increased gut permeability and infiltration of the lamina propria by bacterial metabolites, pollutants, and pathogenic bacteria [130]. Consequently, this dysbiosis, in conjunction with the resulting modulation of the gut immune response and systemic inflammation, could result in the development of several diseases, including T2D, obesity, inflammatory bowel disease, and neurobehavioral dysfunction due to disruption of the gut–brain axis [67].

## 5. Health Impacts of Environmental Exposure

**Figure 4:**
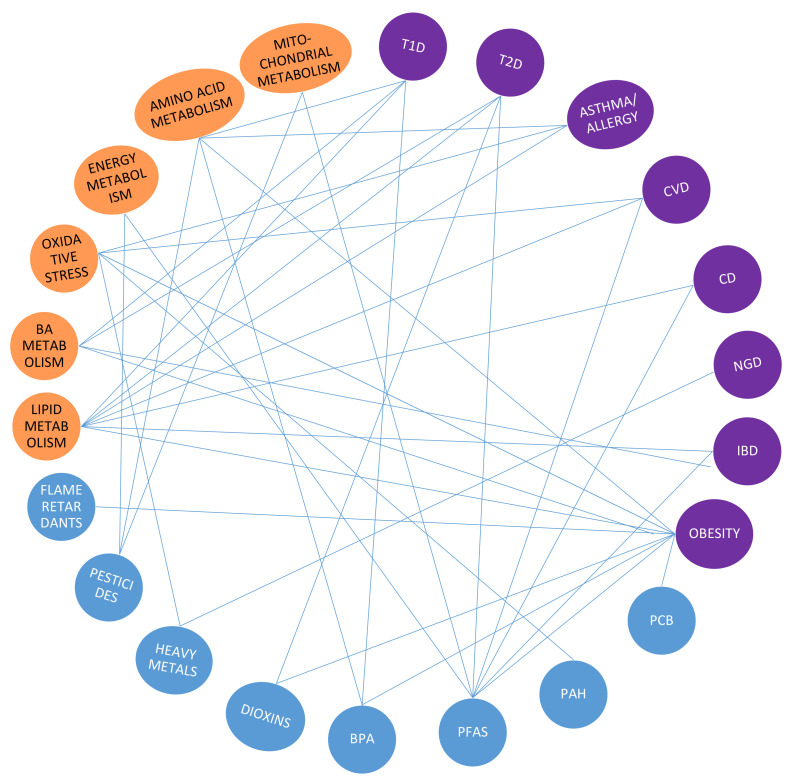
Reported associations between exposure (blue circles), specific diseases (violet circles) and metabolic pathways (orange circles).

There are several studies linking environmental exposure to various diseases, including pre-eclampsia, congenital heart defects, fetal growth restriction, chronic fatigue syndrome, cancer, colonic polyps, respiratory disease, obesity, and diabetes [4,5,26,31,126,131,132,133,134,135,136,137,138,139,140,141,142,143,144,145,146,147,148,149,150,151] (Figure 4). However, it should be emphasized that in most exposome studies, only targeted analyses of specific chemicals have been carried out, and the role of combined exposures as a mixture of multiple chemicals and the metabolome has not yet been systematically studied. Although there are several studies linking metabolic changes with exposure, most of these studies have not characterized the metabolome comprehensively, but instead used clinical metabolic values, such as glucose, insulin, and clinical lipid markers. On the other hand, several metabolic biomarkers have been identified for different chronic diseases. These biomarker profiles could also be used as adverse health outcome markers, when the actual health outcomes are not known. This is particularly important in the cases where disease development is still in its early, prodromal stages, e.g., in prediabetic subjects. Furthermore, understanding the early molecular events along the exposure–disease continuum will provide valuable information that may be used to develop intervention and prevention strategies. As already discussed above, it is also crucial to critically examine if the found exposome-health associations reflect potential causal relationships, or spurious associations.

Below, we review the studies linking chemical exposures, metabolic markers, and selected health outcomes, including as related to early development, metabolic and autoimmune diseases (type 1 diabetes).

### 5.1. Exposures during Early Development

Metabolomics can be utilized to characterize internal exposures or health status during pregnancy. Phthalates, bisphenols and PFAS have been reported to have an influence on childhood growth. Specific lipids, including fatty acid metabolites, amino acids and a steroid have been identified as potential biomarkers of “small for gestational age” during pregnancy, indicating potential dysregulation of lipid pathways in the placenta. However, any association with maternal health status remains unclear [152]. Maternal urinary levels of branched-chain amino acids and steroid hormone by-products were found to be strongly predictive of birth weight [153], while several circulating metabolites have also been associated with birth weight, including lysophospholipids [154,155]. Maternal smoking during pregnancy is known to have several adverse health effects on the offspring, including lower birth weight and other health impacts later in life. Maternal smoking has been associated with alterations of the fetal metabolome, particularly phospholipid profiles [156]. In experimental models, prenatal exposure to BPA caused significant changes in metabolites associated with lipid, BA, amino acid, and glucose metabolism [112,157,158,159]. 

There are several reports on the impact of PFAS exposure during fetal development and early life on the health outcome of children, with PFAS exposure being associated with cardiometabolic risk factors including reduced birth weight, reduced birth length, and increased adiposity. However, the results of such studies have been inconsistent [139,142,160,161]. Whilst many of these risk factors have been associated with alterations in the metabolome, it is still not clear whether the effects of PFAS on health endpoints are mediated by metabolic disturbances. As reviewed recently [162], most studies report association between reduced birth weight and PFAS exposure [163,164,165,166,167], although there are also studies reporting no significant association [168]. It has been suggested that the reduced birth weight could be attributed to maternal lipid changes following on from exposure, as PFOS levels were associated with reduced levels of polyunsaturated fatty acids in pregnant women, and the changes were further associated with reduced birth weight in female infants [169]. Several studies also identified associations of PFAS with increased body weight later in life [135,166,170], as well as with higher insulin levels [171], although contradictory results have also been reported [172]. PFOA levels in childhood were found associated with lower pancreatic β-cell function in adolescence [135]. PFAS exposure in childhood has also been associated with dysregulation of several lipid and amino acid pathways, as well as longitudinal alterations in glucose homeostasis in overweight and obese children [114].

### 5.2. Type 2 Diabetes, NAFLD, Obesity, and Metabolic Syndrome

Several studies in human cohorts have shown association between chemical exposure and T2D [173]. 

A negative association has been observed between serum concentrations of PCB-118, β-HCH and specific PFAS with lobular inflammation in the liver [29,174].

PFAS exposure was found associated with metabolic diseases such as diabetes, being overweight/obese and heart diseases, with the strongest association reported across different studies being dyslipidemia. According to a recent review, there is relatively consistent evidence of modest positive associations between PFAS exposure and lipid profiles, such as total cholesterol and triglycerides, although the magnitude of the cholesterol effect is inconsistent across different exposure levels [175]. A study of Danish children studied the association of PFOS and PFOA with clinical metabolic markers (blood glucose, insulin, triglycerides, adiponectin and leptin), and found that, in overweight children, high PFAS levels were associated with elevated insulin levels, β-cell activity, insulin resistance, and TGs. There was no such association in normal-weight children [148]. Another study, with 74 children with NAFLD and a control group (age 7–19 years) showed that higher PFAS exposure was associated with more severe disease in children with NAFLD, indicating that PFAS may be an important toxicant contributing to NAFLD progression [115].

A study of elderly Swedes found that perfluorononanoic acid (PFNA) was related to prevalent diabetes in a non-monotonic fashion, suggesting an influence on glucose metabolism in humans at the level of exposure seen in the general elderly population. In the same study, PFOA was also associated with insulin secretion, although none of the measured PFASs were associated with insulin resistance [23]. Another study investigated the associations of four PFASs (PFOA, PFNA, PFOS, and perfluorohexane sulfonic acid—PFHxS) with cholesterol, body size, and insulin resistance, identifying positive associations between both PFOS and PFOA with cholesterol, but not insulin resistance or body size [143].

PFAS exposure was found associated with various measures of blood glucose and cholesterol in several studies [23,176]. Using cross-sectional data from 7904 adults (age ≥ 20 years) in the National Health and Nutrition Examination Survey (NHANES), a strong positive association between serum PFOA and diabetes prevalence in men was identified, and the highest PFOA levels were linked with serum total cholesterol in both males and females [176]. In a recent prospective, a nested case–control study in the USA, clinical metabolic markers (cholesterol, triglycerides, adiponectin, HbA1c and insulin) were studied together along with the levels of five PFASs (PFOS, PFOA, PFHxS, PFNA, PFDA) [26]. The results showed that high plasma concentrations of PFOS and PFOA were associated with an elevated risk of T2D. Another study found that PFOS and PFOA were associated with insulin resistance, β-cell function, and HbA1c. After 4.6 years of follow-up, however, these chemicals did not appear to affect the incidence of diabetes or changes in these markers [177].

### 5.3. Type 1 Diabetes

The incidence of several (auto)immune diseases has been increasing in many industrialized countries since 1950s [178]. Among them, the highest increase in incidence of type 1 diabetes (T1D) was observed among children under five years of age [179]. While several non-exclusive hypotheses have been proposed, aiming to explain these incidence trends, their underlying causes are still poorly understood. T1D is an autoimmune disease caused by destruction of insulin-secreting pancreatic β-cells [180]. The strongest genetic risk factors for T1D are found within the human leukocyte antigen (HLA) gene complex, yet only 3–10% of individuals carrying HLA-conferred disease susceptibility develop T1D [162]. The important role of environmental factors, including gene–environment interactions, is thus obvious [181]. For unknown reasons, T1D incidence has stabilized in the last decade, particularly in the Nordic countries [182].

Environmental triggers and specific co-morbidities are often implicated in T1D, e.g., enterovirus infection, diet, and obesity [181]. However, obesity has not shown a concomitant decrease since 2005 [183], and severe enterovirus infections in Finland during the period 2006–2010 increased, in fact, by 10-fold [182]. However, the time trend of human exposure levels to PFOS and PFOA does follow T1D incidence. The use of PFOS and PFOA has increased substantially since production started in the 1950s, until the main, global manufacturer ceased production of PFOS, PFOS-related substances and PFOA between 2000 and 2002. In the EU, all uses of PFOS were banned in 2008.

PFASs are potentially immunotoxic, thus capable of either suppressing the immune system or promoting the development of autoimmune diseases [184]. Recently, the National Toxicology Program reported that, “*PFOA is presumed to be an immune hazard to humans based on a high level of evidence that PFOA suppressed the antibody response from animal studies and a moderate level of evidence from studies in humans*” [185]. Currently, the information on the environmental chemicals as possible triggers of T1D is limited. However, it is plausible that they can contribute to T1D development via impaired pancreatic β-cell and immune-cell function and immunomodulation [133]. It has, for example, been shown that PFOA and PFOS disrupt the generation of human pancreatic progenitor cells [186]. Studies in human cells, i.e., those showing perturbation of the generation of pancreatic precursors caused by PFOA and PFOS, suggest that these compounds might compromise the formation of the mature pancreas, which would result in increased risk for T1D. Moreover, epidemiologic studies have identified a link between exposure to PFOA and PFOS and the occurrence of diabetes, dysfunctions in sugar metabolism, and insulin secretion [135,187]. Epidemiological studies also report immunosuppressive effects of PFAS, increased risk of infection in early childhood and association with immunotoxic effects [139,145]. Recently, elevated levels of PFOS in T1D patients compared to controls was reported [145]. There is a general consensus that exposure to PFOA and PFOS alters the immune system in experimental models, with documented effects including alteration of antibody and cytokine production [188]. Despite this, there remain contradictory studies related to PFAS exposure and T1D and beta-cell autoimmunity, both in epidemiological studies as well as animal models. While most studies have found positive associations between exposure to PFAS and diabetes, autoimmune responses and glucose homeostasis [132,133,135,145], there are also a few studies that show no associations of β-cell autoimmunity and exposure [189], or even a negative association between diabetes and PFAS levels [187]. These contradictory results may be explained by several factors, potentially including differing coverage of PFAS substances, differences in the levels and length of time of exposure in the studies. In vitro and animal models have shown a non-monotonic dose–response of PFAS [132], and this, in combination with differences in, e.g., genetic risk factors, may explain some of the observed differences. Of the other PFASs, PFNA has, in animal studies, been shown to have toxic effects on lymphoid organs, T-cell and innate immune-cell homeostasis, suggesting that these effects may result from the activation of PPARα, PPARγ, and the hypothalamic–pituitary–adrenal axis [190]. In a non-obese diabetic (NOD) mouse model, prenatal and early life exposure to perfluoroundecanoic acid (PFUnDA) was shown to increase pancreatic insulitis (inflammation development, a prerequisite of diabetes development). There was a demonstrable increase in the number of apoptotic cells in pancreatic islets prior to insulitis and decreased phagocytosis in peritoneal macrophages [132,133].

Children progressing to T1D-associated islet autoantibody positivity, or to overt T1D later in life, show a distinct serum lipidomic profile characterized by decreased blood phospholipid levels, including sphingomyelins (SMs), within the first months of life, preceding the onset of islet autoimmunity [191]. These findings, first reported in 2008, have since been confirmed in multiple studies [192], including, most recently, the multinational The Environmental Determinants of Diabetes in the Young (TEDDY) cohort [193]. Notably, these pre-autoimmune lipid disturbances are primarily observed in children who progressed to T1D early, within the first years of life, and such changes can be observed as early as at birth [194,195]. The causes of lipid disturbances in, and their relevance to, T1D pathogenesis have remained elusive. This may have changed recently, given results from a study carried out on a mother–child cohort, where McGlinchey et al. found that high prenatal exposure to PFAS decreases the levels of the same lipids in newborn babies as those previously found to be associated with progression to T1D [34]. The same study also reported the association of PFAS exposure with the onset of islet autoimmunity in children. These findings were confirmed in the DIABIMMUNE cohort, a prospective birth cohort [34]. In the same study, McGlinchey et al. show that high HLA-conferred risk of T1D in infants exacerbated the impact of prenatal exposure to PFAS on postnatal T1D-associated lipid levels [34], suggesting a potentially important and specific role of gene–environment interaction in the development of T1D. Within the same investigation, the causal role of PFAS on postnatal lipid profiles, as well as (previously reported [132,133]) accelerated insulitis, was confirmed in two studies in NOD mice. This study, linking prenatal exposures to PFAS with the postnatal risk of T1D, combined with the aforementioned recent ban on certain PFAS compounds use, may explain the changing trend in the incidence of T1D in certain western countries, thereby also highlighting the need for similar investigations regarding other immune-mediated diseases showing similar incidence trends since the 1950s [178]. The study also exemplifies the MITM approach in exposome research, where chemical exposures are linked to specific adverse health outcomes via intermediate phenotypes such as the metabolome.

### 5.4. Allergy and Obstructive Lung Disease

There is an extensive body of literature on the relationship between environmental exposures and allergic sensitization as well as the causal role in the onset of obstructive lung disease [196,197,198,199,200]. It is not feasible to adequately review this information in the current paper, which is primarily focused on metabolic diseases and diabetes. Instead, we provide a brief summary of the field in relation to the exposome concept [5,201,202]. The lungs are a major route of exposure to the external environment. The average minute ventilation is 6 L per minute under rest and increases as high as 30 L per minute during exercise. This volume of air is a significant potential exposure source. Exposure to air pollutants has been linked with the inception, development, and exacerbations of allergic and pulmonary diseases (in addition to indoor and outdoor aeroallergens) [201]. In particular, traffic-related air pollutants (TRAPs) including nitrogen dioxide (NO_2_), ozone (O_3_), volatile organic compounds, and particulate matter (PM), as well as environmental tobacco smoke (ETS), have been linked with allergic sensitization and asthma [5,203,204,205] as has household air pollution [206]. There are numerous reports on the relationship between exposure to TRAP and ETS with the onset of respiratory diseases and interested readers are referred to recent reviews [207]. However, it should be emphasized in the context of lung diseases that the exposome constitutes more than the collection of air pollutants that are recognized triggers for lung injury. Airborne PM contains diverse populations of bacteria, viruses and fungi that affect respiratory health through infections and modulation of the immune system [208]. A consensus document from the American Academy of Allergy, Asthma, and Immunology (AAAAI) and the European Academy of Allergy and Clinical Immunology (EAACI) reported that the exposomic approach is particularly applicable to allergic diseases and asthma because it provides a risk profile instead of single predictors [209]. The power of an exposomics approach becomes clearer in the context of mixtures-based exposures—for example, simultaneous allergen and diesel exhaust [210] or allergen and phthalates [211,212,213]. 

While air pollutants are a known risk factor for asthma and chronic obstructive pulmonary disease (COPD) [214], their role in allergic diseases in general is not well established [203]. Moreover, the composition of PM has typically not been investigated in this context. PM is composed of a complex mixture of both inorganic (e.g., trace elements) and organic compounds (e.g., PAHs, alkanes), and the composition can be variable, depending on the source. Thus, linking only the amount of PM with specific health outcomes may not give a realistic picture of the possible associations. Currently, there are relatively few clinical cohort studies reported on the relationship between exposome–metabolome allergy. A recent European Human Early-Life Exposome cohort study found no association between any of the broad spectrum of childhood environmental studies and allergy-related health outcomes, while prenatal exposure to mono-4-methyl-7-oxooctyl phthalate was associated with an increased risk of rhinitis, whereas PM absorbance was associated with a decreased risk [215]. Exposure to phthalates has been associated with decreased risk of eczema in some studies [216], but not others [217]. It should be noted here that phthalates have a short half-life and the concentrations will therefore be variable, plausibly explaining the inconsistent findings reported in the literature. The European Food Safety Authority (EFSA) reported that PFAS exposure has showed no or inconsistent associations with asthma and allergies for both prenatal and postnatal exposures as well as in children and adults [218]; however, a recent study with the (National Health and Nutrition Examination Survey) NHANES cohort reported a weak association between serum PFAS levels and asthma prevalence in children [219]. A recent metareview reported that perfluorononanoic acid was associated with eczema, perfluorooctanesulfonic acid with atopic dermatitis and perfluorooctanoic acid with allergic rhinitis, while no significant associations were found for wheeze and asthma [220].

A number of specific compounds have been linked with increased risk of food allergy [221]. For example, the antibacterial agent triclosan has been positively associated with food and aeroallergen sensitization in male children in the NHANES cohort [222]; however, neither a Norwegian study nor a study from the USA observed an association between triclosan exposure and food allergen sensitization [223,224]. A few studies have examined the impact of phthalate exposure and have found that it is associated with an increased risk for food sensitization in children [225,226]. Given the increasing incidence of food allergies [227,228], this will be an important area of future study. 

Given that obstructive lung disease and allergy have low genetic determination [229], there is a vital role for application of the exposome concept to increase our understanding of disease etiology. It is expected that exposome investigations will provide insight into the role that complex environmental mixtures exert in the onset and pathophysiology of allergic sensitization and pulmonary disease. However, as with other exposome-based studies, it will be important to ensure proper study design and to examine, in particular, the role of pre-natal exposures. 

## 6. Conclusions

The applications of metabolomics to exposome research, aiming to link external exposures with adverse outcome pathways and health outcomes, are an increasingly active area of research [67]. Although already being addressed in current research, two main challenges in exposome research still remain:

*1. Analytical coverage.* Given the enormous complexity of the chemical exposome, and the vast number of chemicals to consider, at concentration ranges covering several orders of magnitude, analysis of these chemicals alongside the metabolome is challenging at the very least. One limitation, particularly in human cohort studies, is also the amount (physical volume) of sample available for the analysis, which limits the number of different analytical methods that can be applied to the same sample. This favors analytical methods with broad analytical coverage, yet this often leads to inevitable tradeoffs in terms of accuracy of quantification as well as sensitivity. 

*2. Data integration and establishing causal relationships between exposures and adverse health outcomes.* As also shown in this review, a large number of studies have established associations between specific chemical exposures and metabolic outcomes (i.e., clinical metabolic markers or metabolomics) or specific adverse health outcomes. However, in most cases, the question remains if these are true causal relationships or associations confounded by other factors such as diet. This challenge is likely to become even greater with increasing analytical coverage of the chemical exposome and metabolome, and with the inclusion of other data such as from the gut microbiome. Establishing causality is crucial if one is to consider the safety of specific chemicals or specific prevention measures. The elimination of spurious associations (e.g., by a rejection-rate-filtered partial correlation network approach), the identification of key toxic drivers (e.g., by regression of selected chemicals with selected outcomes of interest), and follow-up with targeted exposure studies in relevant experimental models [34], are likely to be key research strategies suitable for tackling the challenge of data integration and proving causality in exposome research.

Given the active research in the exposome field, it is likely that the future will bring many innovative solutions to address the above challenges. Such advances will have the potential to open new areas of investigation related to the study of the impact of real-world chemical exposures on human health and for more accurate chemical safety assessment, as well as challenge our current views about the origin and pathogenesis of many common diseases.

## Abbreviations

12-oxo-LCA	12-Oxolithocholic acid
7-oxo-DCA	7-Oxodeoxycholic acid
7-oxo-HCA	7-Oxohyocholic acid
ANN	Artificial neural networks
AT	Adipose tissue
BA	Bile acids
BAAT	Bile acid CoA: amino acid N-acetyltransferase
BACS	Bile acid-coenzyme A synthase
BADGE	Bisphenol A diglycidyl ether
BPA	Bisphenol A
BSE	Bile salt export pump
CA	Cholic acid
CDCA	Chenodeoxycholic acid
CE	Cholesteryl ester
Cer	Ceramide
CYP7A1	Cytochrome P450 family 7 subfamily A member 1
DCA	Deoxycholic acid
DDA	Data-dependent acquisition
DDE	Dichlorodiphenyldichloroethylene
DES	Diethylstilbestrol
DG	Diradylglycerol
DHCA	3α,7α-Dihydroxycholestanoic acid
DIA	Data independent acquisition
EFSA	European Food Safety Authority
FT-ICR	Fourier transform ion cyclotron resonance
FXR	Farnesyl-X-receptor
FXR	Farnesoid X factor
GC	Gas chromatography
GCA	Glycocholic acid
GCDCA	Glycochenodeoxycholic acid
GDCA	Glycodeoxycholic acid
GDHCA	Glycodehydrocholic acid
GHCA	Glycohyocholic acid
GHDCA	Glycohyodeoxycholic acid
GLCA	Glycolithocholic acid
GUDCA	Glycoursodeoxycholic acid
HCA	Hyocholic acid
HCB	Hexachlorobenzene
HDCA	Hyodeoxycholic acid
HLA	Human leukocyte antigen
HNF4a	Hepatocyte nuclear factor 4 alpha
HRMS	High-resolution mass spectrometry
IT–TOFMS	Ion trap–time-of-flight mass spectrometry
LCA	Lithocholic acid
LPC	Lysophosphatidylcholine
L-PFOS	Linear-perfluorooctane sulfonate
MITM	Meet-in-the-middle
MnBP	Mono-n-butyl phthalate
NAFLD	Non-alcoholic fatty liver disease
NO2	Nitrogen dioxide
NTCP	Sodium taurocholate co-transporting polypeptide
O3	Ozone
OSTα/β	Organic solute transporter α/β
PAH	Polyaromatic hydrocarbons
PAH	Polycyclic aromatic hydrocarbons
PBDE	Polybrominated diphenyl ether
PC	Phosphatidylcholine
PC ethers	Phosphatidylcholine ether
PCB	Polychlorinated biphenyls
PE	Phospatidylethanolamine
PFAS	Per- and polyfluoroalkyl substances
PFBA	Perfluorobutanoic acid
PFBS	Perfluorobutane sulfonate
PFDA	Perfluorodecanoic acid
PFDS	Perfluorodecane sulfonate
PFECHS	Potassium perfluoro-4-ethylcyclohexanesulfonate
PFHpA	Perfluoroheptanoic acid
PFHpS	Perfluoroheptane sulfonate
PFHxS	Perfluorohexane sulfonate
PFNA	Perfluorononanoic acid
PFNS	Perfluorononane sulfonate
PFOA	Perfluorooctanoic acid
PFOSA	Perfluorooctane sulfonamide
PFPeA	Perfluoropentanoic acid
PFPeS	Perfluoro pentane sulfonate
PFTDA	Perfluorotetradecanoic acid
PFTrDA	Perfluorotridecanoic acid
PFUnDA	Perfluoroundecanoic acid
PI	Phosphatidylinositol
PM	Particulate matter
POP	Persistent organic pollutants
ROS	Reactive oxygen species
SM	Sphingomyelin
T1D	Type 1 diabetes
T2D	Type 2 diabetes
TBT	Tributyltin
TCA	Taurocholic acid
TCA	Tricarboxylic acid cycle
TCDCA	Taurochenodeoxycholic acid
TDCA	Taurodeoxycholic acid
TDHCA	Taurohyodeoxycholic acid
TG	Triradylglycerol
THCA	Taurodeoxycholic acid
THDCA	Taurohyodeoxycholic acid
TLCA	Taurolithocholic acid
TRAP	Traffic-related air pollutants
TUDCA	Tauroursodeoxycholic acid
TαβMCA	α,β-Tauromuricholic acid
TωMCA	ω-Tauromuricholic acid
UDCA	Ursodeoxycholic acid
UGT	UDP-glucosyltransferases
UHPLC	Ultra-performance liquid chromatography
β-HCH	β-hexachlorocyclohexane
βMCA	β-Muricholic acid
ωαMCA	ω-Tauromuricholic acid
